# Fibre post behaviour prediction factors. A review of the literature

**DOI:** 10.4317/jced.50619

**Published:** 2013-07-01

**Authors:** Elisa Bru, Leopoldo Forner, Carmen Llena, Amelia Almenar

**Affiliations:** 1 DDS. Department of Stomatology. Universitat de València. Valencia, Spain; 2 MD, DDS, PhD. Professor. Department of Stomatology. Universitat de València. Valencia, Spain; 3 MD, DDS, PhD. Department of Stomatology. Universitat de València. Valencia, Spain

## Abstract

Introduction: The advantages of fibre posts over stainless steel posts have been demonstrated in numerous studies. For clinical success, various factors need to be taken into account in post-retained restorations. Material and Methods: A review was made of literature on fibre posts published from 2000 to February 2011, identified through searches of the PubMed/Medline databases. Results: The position of the tooth in the arch, ferrule size, proximal contact, periodontal support and restoration type are survival prediction factors that should be considered when performing post-endodontic restoration. Since fibre posts present good biomechanical behaviour as a result of their elastic modulus, which is similar to that of dentine, treatment failure occurs through decementation rather than because of root fracture, as occurs with metal posts. The shape of the post and, consequently, the thickness of the luting, can modify the retention capacity. Consequently, more anatomically-shaped posts have been developed, as have new techniques to reduce the volume of the dentine/cement interface: lateral condensation, surface remodelling or custom designs. Conclusions: Different aspects of the preparation process can be modified to assist in improving fibre post retention, but further investigation, mainly clinical, is needed to acquire a better understanding of how different factors influence the long-term clinical behaviour of the posts.

** Key words:**Fiber post, post shape, post adaptation, post retention, endodontics.

## Introduction

Conservative dentistry aims to preserve as much of the tooth structure as possible or at least to retain the tooth in the mouth. When the tooth has lost a large proportion of its structure, auxiliary means are required to ensure retention of the filling material. Intraradicular posts are used for this purpose in teeth that have undergone root canal treatment (1).

The advantages of fibre posts rather than stainless steel models have been proved by numerous studies which have demonstrated the clinical success of fibre post systems implanted by adhesive techniques. This success is due to their presenting good retention properties under mechanical strain as a result of their low elastic modulus, which is similar to that of dentine: 18-42 Gpa (2,3). This biomechanical behaviour avoids the appearance of root fractures (4-6). Nonetheless, insufficient post rigidity could lead to excessive deformation and stress concentration zones during function, giving rise to marginal failure (7,8).

A number of factors can influence the survival of intraradicular post-retained restorations. Firstly, there are dental factors such as how much of the tooth remains or whether there is a ferrule measuring at least 1.5-2 mm in length (8), and conditions such as periodontal status, relation to adjoining and opposing teeth, parafunction or habits that may constitute contraindications for tooth restoration. Secondly, there are post-related factors: length, taper, diameter and the material from which the post is made (9). Nowadays a wide range of posts is available: round or oval, with different degrees of taper (10), more or less translucent to improve curing light transmission and made of different materials: fibreglass, carbon fibre, quartz fibre, silica or hybrids. Also, surface integrity is indispensable for the fibre posts to adhere to the composite cement (7). Lastly, there are cement-related factors: curing shrinkage, difficulties for the curing light to reach apical zones, chemical incompatibility with the adhesive system, and the moisture control required to apply the adhesive system (11,12). An excessively thick layer of luting around the post has been associated with a high frequency of post decementation due to increased pores and spaces between the cement and the root dentine (10), but the thickness and contraction of the luting depends on the root area and the shape and material of the post employed.

Because of all these factors, a wide variety of posts has been designed with the aim of achieving a retaining element with optimal retentive, biomechanical and aesthetic properties.

This paper reviews the current position of research into fibre posts, focusing on the factors that influence their clinical performance.

## Material and Methods

A bibliographic review was made of articles on fibre posts published from 2000 to February 2011, identified through searches of the PubMed/Medline databases. The search terms employed were: fiber post, post shape, post adaptation, post retention, push-out test, radicular dentin preparation. The types of document included articles on both in vitro and in vivo research, prospective and retrospective studies and reviews on the subjects of luting, post shape and surface, influence on restoration retention and risk and survival factors.

## Risk factors that do not depend directly on post type

In a prospective study published in 2005, 144 posts were followed up over a five-year period. The failure rate was 6-7% (13). In another study of the same type dating from 2008, the rate in 149 posts was 6.6% a year (14). Post fracture was the most frequent cause of failure, followed by loss of retention. Incisors and canines presented far higher failure rates than posterior teeth, as the occlusal force is much more transverse in anterior teeth than in posterior teeth, where it is more axial (13,14). A high number of vertical fractures is found in maxillary premolars. This could be related to the small mesio-distal diameter of the canal, which could favour stress within the root, especially when good post/dentine adhesion is not achieved (15). Root fracture rates of 2-5% are cited for stainless steel posts, where the loading stress is much higher (5): 803 N compared to 500 N for fibre posts (6).

The number of surfaces preserved is an indicator of restoration survival. Teeth with fewer than three surfaces have 1.8 times more failures than teeth with three or more surfaces. The presence of 2 mm of ferrule increases resistance to fracture (14). Also, teeth with no proximal contact fail almost three times more often than those with proximal contact (13,14).

As regards restoration type, a combination of fixed dental prostheses and removable prostheses increases the failure rate significantly. This is followed by single crowns, while fixed bridges have the highest survival rate. The crown protects the tooth and eliminates the effect of diameter in stainless steel posts, but it does not completely exempt the tooth from root fracture (5)

Other factors that have to be taken into account for post survival involve occlusal contact, tooth position in the arch, crown position and periodontal support, which are all related to the way that forces are loaded and the tooth’s ability to assimilate them, and also to the amount of dentine remaining, as the ferrule effect is decisive for survival (14).

## Luting thickness and post shape

With stainless steel posts it has been found that the greater the post diameter, the lesser the force required to bring about root fracture, whereas the diameter of fibre posts does not have a significant influence on biomechanical performance (fracture load and stress distribution). This is because the elastic modulus of fibre posts is similar to that of dentine. Post length does not influence biomechanical behaviour in either case. Consequently, the restoration technique is less sensitive to post dimension in the case of fibre posts than when stainless steel posts are employed (5).

An excessively thick layer of luting around a fibre post is an unfavourable factor for the long-term success of post-retained restorations, owing to the high frequency of decementation (3,10). Passive retention of the post is improved if it fits snugly into the prepared space and if the luting layer is fine and even (3,4). Post decementation generally occurs at the dentine/cement interface (12,16) due to the bubbles and pores that form as a result of curing stress (4). The bond between the luting and the radicular dentine is sometimes unable to withstand curing shrinkage (11). The luting adhesion strength varies along the length of the root canal. It is greater in the cervical area, because polimerization is less successful in reaching the root areas, which are also where more debris (gutta-percha and smear layer) is found and where moisture control is more complicated (11). However, if the post fits well coronally there is less likelihood of decementation (3,17). If the cement adheres to the post when it is removed, the post can be reused, as its shape will fit that of the canal and be more suitable for recementing (17). On reproducing the biomechanical behaviour of maxillary incisors restored with fibre posts, it was observed that the debonding occurred in the vestibular area along the interface with the post, making it possible to repair the root in question (5,6).

A comparison of oval and round section fibre posts, preparing the former with oval-section medium and fine grain ultrasonic diamond tips and the latter with a specific drill for this type of post, observed that the canals prepared with fine ultrasonic tips that received oval posts showed the lowest cement thickness in the apical third and similar thicknesses in each third, while the round posts presented greater cement thickness but preserved more root dentine (10). Another study of anatomical and standard posts showed significantly lower cement thickness in the middle and coronal thirds with the anatomical posts, but no significant difference apically between the groups (17). In oval section root canals, both circular and oval fibre posts achieved similar retention strengths. Neither the post shape (with its respective cement thickness) nor the luting cement material had a significant effect on bond strength (push-out) (15). The use of oval points rather than round points to fill oval canals has the advantage of providing a cleaner space for the post, whereas instrumenting with rotary nickel-titanium instruments tends to create a central hole that sacrifices healthy tissue and weakens the root more (10,15,18).

Luting thickness is not a factor that influences bond strength, according to another study which assessed luting thickness with cylindrical fibre posts of two different sizes. The push-out tests showed no significant differences in bond strength, but did find statistically-significant differences in luting thickness (12).

However, when the effect of luting thickness on bond strength was studied in order to obtain a thickness measurement that would favour adhesion, it was found that luting thickness did affect fibre post bond strength. With the posts employed (quartz fibre), the greatest bond strength values were achieved with luting thicknesses of 0.1-0.3 mm. With higher or lower luting thicknesses, post retention decreased (4).

Although the continuity of the cement (or, conversely, the number of gaps) varies according to the materials used and increases when teeth are kept in water, this does not alter post retention. However, the water swells the cement and pushes it against the walls, so the post is retained more by friction than adhesion. Friction is the main cause of post retention (11).

## Treatment of post surfaces and dentine

Post surface treatments (sandblasting and silanization) can improve post/cement adhesion, but this does not translate into better retention because the cement has limited adherence to the radicular dentine (9).

To create adhesion, it is essential to dry the radicular dentine before cementing in the post. Ethanol can assist in improving drying but does not improve post adhesion (19), whereas acid etching of the root dentine is essential for good adhesion of resin cement. The post retention results are better after 15 seconds of acid etching than after 30 seconds (20). Irrigation with 2% chlorhexidine following acid etching, before cementing the post, reduces cement/post bond failures significantly and slightly improves bond strength for almost all the post systems examined (16).

Laser treatment of the dentine surface to improve dentine/post adhesion has also been studied, but in one study the use of a Nd:YAG laser had a negative effect on post retention strength (21).

## Fitting the posts to the anatomy of the canal

In the case of very wide canals that present a risk for post retention, an alternative method for reducing the discrepancy in shape between the canal and the post is pseudo-lateral condensation, using a main post and several small auxiliary posts (22). The fracture resistance of teeth reconstructed with standard posts of various sizes and their combination with several small posts has been studied. Fracture resistance for posts in the 0.82 and 0.98 mm (apical diameter) sizes was similar to that for a combination of small posts. The greatest resistance was obtained with a 1.37 mm (apical diameter) post. Consequently, single large-diameter posts appear to be a better option than a combination of several small posts for large round canals such as those of canines, whereas using several posts would be a good solution for oval canals where standard posts do not give a good fit. Both reconstruction methods would be suitable for other canal types (22).

Remodelling the surface of fibreglass posts to create custom posts with a similar shape to that of the canal modifies the diameter of the posts without loss of surface integrity, as the resulting surface does not differ from that of commercial fibre posts (7). The procedure is to take a cast of the interior of the canal and use it as a model, copying it by cutting the post with a diamond bur.

Another individual post fitting system is to use a fibreglass mesh, inserting it into the canal to act as the post. A study of this technique showed that the number of gaps observed with a scanning electron microscope was greater with this system than with prefabricated posts (23).

## Conclusions

The prognosis for fibre posts is better in posterior teeth than in anterior teeth. A larger tooth remnant and a greater number of proximal contacts also improve the prognosis for post-endodontic restorations.

The main cause of fibre post failure is decementation at the cement/dentine interface, whereas with metal posts failure mainly occurs as a result of excessive stress on the dentine, giving rise to root fractures. The mechanical properties of fibre posts are similar to those of dentine, so little stress arises during function and the stress is evenly spread all along the root.

Luting thickness shows no influence on post bond strength. Post surface treatments do not have any great effect, unlike dentine surface preparation when using adhesive techniques.

The different procedures for attempting to improve the fit between posts and noncircular root canal walls do not show greater efficacy.

Further studies, mainly clinical, are required to acquire a better understanding of the effects of the different factors that affect the long-term clinical retention performance of intraradicular posts.
